# The pH and Sucrose Influence Rhamnolipid Action Toward Planktonic and Biofilms of *Listeria monocytogenes*

**DOI:** 10.3390/microorganisms12102078

**Published:** 2024-10-17

**Authors:** Tathiane Ferroni Passos, Marcia Nitschke

**Affiliations:** São Carlos Institute of Chemistry (IQSC), University of São Paulo, Trabalhador São-Carlense Av., 400, P.O. Box 780, São Carlos 13566-590, SP, Brazil; tathianefp@gmail.com

**Keywords:** biosurfactant, biofilm, Listeria, micelle, pH, rhamnolipid, sucrose

## Abstract

Bacterial resistance and persistence in food environments are major concerns for the industry, which constantly seeks new strategies to reduce microbial contamination. Rhamnolipids (RL) biosurfactants are considered sustainable and green alternatives to synthetics; furthermore, they have demonstrated potential for controlling various foodborne pathogens. Food environments are typically exposed to diverse pH, solutes, temperatures, and water activity (a_w_) levels that may favor the survival of pathogens. Therefore, it is crucial to consider these factors in evaluating the performance of novel antimicrobials. Our study examined the influence of pH and sucrose on the antimicrobial activity of RL against both planktonic and biofilm of *Listeria monocytogenes*. We found that the presence of sucrose can enhance the antimicrobial effectiveness of RL against both planktonic and sessile bacteria. The addition of sugar particularly improved RL action at pH 6 and 7. Moreover, we observed that the type and size of RL self-assembly structures depend on the pH and sucrose concentration. These findings suggest potential for developing RL-based innovative methods to control *L. monocytogenes* in sugar-rich or -low a_w_ foods and environments.

## 1. Introduction

*Listeria monocytogenes* is a Gram-positive bacteria that is ubiquitous in nature; hence, it can easily contaminate fresh and processed foods and environments. The consumption of food contaminated with such bacteria can cause listeriosis, a severe foodborne disease (FBD) showing a high mortality rate [1]. Listeriosis is the third cause of death from FBD in the USA, and a recent report estimated that the number of cases can reach 1600 per year, resulting in 260 deaths [2]. Numerous outbreaks associated with *L. monocytogenes* infection have been reported worldwide. More recently, deli-sliced meat contaminated with *L. monocytogenes* caused 57 hospitalizations and nine deaths in the USA [3]. In 2022, the European Union confirmed 2770 cases of listeriosis [4], and a prolonged outbreak (2012–2024) related to the consumption of smoked fish contaminated with *L. monocytogenes* ST173 strain caused 14 deaths in EU countries [5]. Listeriosis cases are sub-notified in Brazil; however, among 2320 food samples analyzed from 2011 to 2021, *L. monocytogenes* was detected in 48.9% of meat products and 36.5% of vegetables [6]. Therefore, the presence and persistence of *L. monocytogenes* in the food chain is of great concern for public health.

The notable ability of *L. monocytogenes* to adapt under stressful conditions such as acidity, low temperatures, and low water activity [7], together with their aptitude to establish biofilms [8], is responsible for their persistence in food environments and subsequent contamination of products.

In order to ensure food safety, it is crucial to effectively control *L. monocytogenes*; thus, food processors actively seek novel methods to guarantee the innocuity of their products. Moreover, the preference for natural and green food additives to replace synthetic ones stimulates the demand for healthier and sustainable alternatives [9].

Surface-active compounds synthesized by microorganisms also known as biosurfactants (BSs) have demonstrated useful properties such as biodegradability, low toxicity and stability toward pH, ionic strength, and temperature, which can be advantageous for industrial applications. Moreover, BS production from renewable substrates contributes to sustainability and circular bioeconomy [10,11] turning BS prospective candidates for innovation. Indeed, traditional surfactant manufacturers are offering their bio-based counterparts to a growing market projected to reach USD 2.3 billion by 2028 [12].

Rhamnolipids (RLs) are well-known microbial-derived surfactants usually synthesized by species of the genus *Pseudomonas.* The structure of RL comprises a glycolipid-type BS that is naturally produced as mixtures of homologs containing one or two rhamnose molecules linked to β-hydroxy fatty acids with different chain lengths [13].

The literature has reported the use of RLs in several industrial fields, including bioremediation, oil recovery, cosmetics, pharmaceuticals, agriculture, and food [14]. Food application is primarily focused in surfactant and emulsifier properties of RL; however, most studies emphasize their remarkable biological activity [15,16,17]. In fact, RLs have shown effective antimicrobial action toward various foodborne bacteria, both planktonic and biofilms [16,18].

A screening study using 32 *Listeria monocytogenes* isolates demonstrated that 90% were susceptible to RL, showing MIC from 78.1 to 2500 µg/mL; in addition, RL showed a synergistic effect with nisin [19]. Another study revealed that the antibacterial action of RL is pH-dependent and more effective under acidic conditions. According to the authors, the dissociation of carboxyl groups present in RL structures is responsible for the pH-driven activity once they can behave as non-ionic surfactants at low pH, favoring contact with cell membrane targets [16].

The presence of solutes, such as ionic salts, influences physicochemical properties and molecular aggregation of RL [20]. Recent work showed that the addition of 5% NaCl to the medium enhanced the antimicrobial activity of RL against *L. monocytogenes* [21]. Thus, along with pH, the RL activity seems to be influenced by solutes. In this view, RL can be exploited as an antimicrobial in salted food/environments but may also be applied to sugar-rich foods.

A combination of RL and mild heat (60 °C) in low a_w_ (0.92) model systems displayed synergistic effects enhancing the inactivation of *Listeria monocytogenes* Scott A [22]. These results open novel opportunities for the development of an RL-based hurdle approach to control pathogens in low-a_w_ foods, reinforcing the importance of studying the impact of solutes in RL activity. Furthermore, there is no report in the literature regarding RL–sugar interaction and its effect on antimicrobial activity.

Once food environments are exposed to pH changes, the presence of solutes, and low water activity that may favor the persistence of pathogens, it is imperative to consider such factors to evaluate the performance of novel antimicrobial candidates. In this sense, our work described the combined effect of pH and sucrose on the antimicrobial action of RL against planktonic and biofilms of *Listeria monocytogenes*. The influence of the pH-sucrose combination in the molecular aggregation of RL and their relation with antimicrobial activity were also investigated.

## 2. Materials and Methods

### 2.1. Rhamnolipids (RL)

Commercial rhamnolipid aqueous solution 25% (Rhamnolipid Inc.^®^, St. Petersburg, FL, USA) containing approximately 54.4% RhaC_10_C_10_ and 24.2% Rha_2_C_10_C_10_ as the main components was utilized.

### 2.2. Microorganism and Inoculum Preparation

*Listeria monocytogenes* ATCC 19,112 was transferred from frozen stock (−20 °C) to a Tryptic Soy Agar (Acumedia, Lansing, MI, USA) supplemented with 6 g/L of yeast extract (TSYEA) plate and incubated at 37 °C for 24 h. Selected colonies were transferred to TSYE broth, and optical density (OD) was adjusted to 0.10 (610 nm, Genesys 10UV, Thermo Scientific, San Jose, CA, USA), corresponding to approximately 1 × 10^8^ CFU/mL. To conduct the antimicrobial tests in planktonic form, after adjusting the OD, the suspension was diluted 10 times in TSYEB to obtain approximately 1 × 10^7^ CFU/mL [16].

### 2.3. Determination of Minimum Inhibitory (MIC) and Minimum Bactericidal (MBC) Concentrations

The broth microdilution technique was performed according to Clinical and Laboratory Standards Institute [23] guidelines. The TSYEB was prepared with increasing sucrose concentrations (0, 5%, 10%, 25%, and 50% *w*/*v*), and the pH values were adjusted between 5.0 and 8.0 using 0.1 M HCl or NaOH solutions. The final medium, adjusted to each pH, was sterilized by filtration (0.22 μm) prior to being added to the microplate. The RL was diluted in the appropriate medium and serially diluted from 2500 mg/L to 4.9 mg/L. Positive and negative controls were also included. An aliquot of 20 µL of standardized inoculum (as in Section 2.2) was added, and after 24 h of incubation at 37 °C, the lowest concentration where no apparent bacterial growth was observed, was designated as the MIC. To confirm the MIC value, 20 µL of a 1 mg/mL MTT solution (Thiazolyl Blue Tetrazolium Bromide 98%-Sigma^®^) was added to each well, observing the result after one hour. To determine the MBC, the content of each well that presented no apparent bacterial growth in the MIC test was transferred to a TSYEA plate and incubated at 37 °C for 24 h. MBC value was designated as the lowest concentration of RL, which, after the incubation period, did not show microbial growth.

### 2.4. Bacterial Growth Curve

*L. monocytogenes* growth curves were carried out using a fixed concentration of RL (650 mg/L in TSYEB) with 0 and 50% sucrose (*w*/*v*) at pH 5.0, 6.0, 7.0, and 8.0. The growth kinetics of the bacterial population in the presence of RL and the RL–sucrose combination were conducted at different time intervals (0 to 24 h). After each pre-established period, 100 µL of the sample was transferred to a microtube with 900 µL of a saline solution and serially diluted. The number of viable cells was determined using the drop method [24].

### 2.5. Antimicrobial Activity in Bacterial Biofilms

*L. monocytogenes* culture was standardized to approximately 1 × 10^8^ CFU/mL in TSYEB with 0 and 50% sucrose (*w*/*v*) at pH 5.0, 6.0, 7.0, and 8.0. Polystyrene test samples (1 cm × 1 cm × 0.1 cm) were cleaned, sterilized using UV radiation, and placed in the bottom of a 24-well microplate. Bacterial inoculum (1 mL) prepared in each tested condition was added to the surface of the samples and incubated for 24 h at 37 °C to establish the biofilms [25]. To evaluate the antibiofilm activity, RL was diluted in TSYEB at 650 mg/L with 0 and 50% *w*/*v* sucrose, adjusted to each pH value. Further, the 24 h old biofilms were gently washed and transferred to a new microplate, followed by the addition of 1 mL of RL treatments. After 24 h of incubation at 37 °C, the samples were washed and transferred to tubes containing 2 mL of saline solution. Ultrasound (Unique, UltraCleaner 1400A 50/60 Hz, Indaiatuba, SP, Brazil) was applied during 15 min to disaggregate the biofilm. Serial dilutions were performed, and viable cells were quantified using the drop method [24].

### 2.6. Determination of Minimal Biofilm Inhibitory and Minimal Biofilm Eradication Concentrations (MBIC) and (MBEC)

Biofilms of *L. monocytogenes* were established in 96-hole peg lid microplates (Nunc-TSP, Thermo Scientific, San Jose, CA, USA) according to the method previously described [26]. The inoculum was adjusted to 1 × 10^8^ CFU/mL in TSYEB, and the peg lid plate was inoculated with 160 µL of the broth and incubated for 4 h at 37 °C. Then, the lid was transferred to a new microplate containing 180 µL of TSYEB with 0 and 50% sucrose at pH 5.0 and 7.0 and incubated at 37 °C, 80 rpm (TE-420 Incubator Tecnal, Piracicaba, SP, Brazil) for 24 h. After biofilm formation, the peg lid was transferred to a treatment microplate containing a serial dilution of RL in each condition of sucrose/pH and incubated at 37 °C for 24 h. The peg lid was then washed twice (200 µL) with saline solution, transferred to a recovery microplate containing TSYEB (200 µL) and, to disaggregate the biofilm, the plate was submitted to the ultrasound (Unique, UltraCleaner 1400A 50/60 Hz) for 10 min. To determine MBEC, the peg lid was kept in the recovery microplate for 72 h at 37 °C, and the MBEC value was designated as the lowest concentration of RL, which, after the incubation period, did not show microbial growth.

### 2.7. Critical Micellar Concentration (CMC)

The effect of sucrose and pH in the aggregation behavior of the biosurfactant was determined using 650 mg/L aqueous solutions of RL, adjusted to increasing concentrations of sucrose and variable pH. The surface tension of each solution was measured using the Du Nouy ring method with a tensiometer (Sigma 700, Attension, Espoo, Finland). CMC values were calculated using the Attension Sigma Software (One Attension v.1 access date on 14 October 2024) considering a maximum standard deviation (SD) of 0.10 mN/m.

### 2.8. Assessment of Molecular Aggregates of RL

To visualize micellar structures of RL, ethanolic solutions of biosurfactant and Nile Red dye (1000 mg/L) were prepared. An aliquot of 700 µL of the RL solution was mixed with 1 µL of dye and centrifuged at 800 rpm (Mini spin centrifuge, Eppendorf AG) for 30 min. Ethanol was further evaporated to eliminate excess dye [27].

#### 2.8.1. Fluorescence Microscopy

The RL/Nile red samples were dissolved in 100 µL of aqueous solution containing 0.5% NaCl and 0.25% KH_2_PO_4_ at pH values adjusted to 5.0 to 8.0 added with 0 and 5% of sucrose. The morphology of the RL aggregates was immediately observed with Fluorescence Microscopy (Labomed, iVu 7000, Jenoptik, Jena, Germany) using a 535 nm filter at 40×. Images were generated using the system software (ProgRes Capture Pro v. 2.10.0.1).

#### 2.8.2. Cryo-TEM

The morphology of RL molecular aggregates was also observed with cryo-transmission electron microscopy (Cryo-TEM). Samples were prepared as above (2.8) and suspended in aqueous solutions containing 0 and 5% of sucrose at pH values adjusted to 5.0 and 7.0. In the same way, the behavior of the RL aggregates was evaluated in the presence of *L. monocytogenes* cells. The inoculum was adjusted to 1 × 10^8^ CFU/mL (Section 2.2) and centrifuged at 10,000 rpm for 10 min (Mini spin centrifuge, Eppendorf, Hamburg, Germany). The cell pellet was further suspended and mixed (1:1) in each RL solution. The samples were mounted in a Vitrobot Mark IV system (Thermo Fischer Scientific, Waltham, MA, USA) equilibrated at 22 °C and humidity 100%, using a TALOS F200C (Thermo Fischer Scientific, Waltham, MA, USA) electron microscope operating at 200 kV. The images were acquired with a CMOS camera, Ceta 16 M (Thermo Fischer Scientific, Waltham MA, USA). Sample preparation and data acquisition were performed at the Electron Microscopy Laboratory (LME)/Brazilian Nanotechnology National Laboratory (LNNano).

### 2.9. Dynamic Light Scattering (DLS)

RL solutions (650 mg/L) were prepared in ultrapure water containing 0.5% NaCl and 0.25% KH_2_PO_4_ with 0 and 50% sucrose, and pH was adjusted from 5 to 8. The samples were filtered (0.45 µm), and the size and distribution of the structures were determined using DLS equipment (Nano Zetasizer, Malvern, UK) at 25 °C, with a dispersion angle of 173°. Each sample was analyzed with at least 10 replicates at four independent moments, and the polydispersity index (PDI index) was calculated using the equipment’s software (ZSExplorer v. 1).

### 2.10. Statistical Analysis

MIC, MBC, MBIC, and MBEC values were expressed as the mode of at least three independent replications. For the other antimicrobial assays, at least three independent replicates were also performed, and the values were reported as the mean and their respective standard error obtained using Origin software (Origin Lab Corporation, Northampton, MA, USA, 2020).

## 3. Results and Discussion

### 3.1. Antimicrobial Activity in Planktonic Cells

*L. monocytogenes* ATCC 19112 was previously selected as the model strain for this work once it was able to grow under the range of pH and solute concentrations proposed. To study the influence of pH and sugar in the antimicrobial action of RL toward *L. monocytogenes*, the MIC tests were performed using culture broth previously adjusted to each pH and sucrose concentration. We observed that both the increase in the sucrose concentration and the variation in the pH of the medium had an impact on the antimicrobial activity of RL (Table 1). In the absence of sucrose, the variation of the pH from 5.0 to 8.0 resulted in an increase in the MIC from 19.5 mg/L to >2500 mg/L. However, the addition of sugar showed a distinctive effect as the pH of the medium increased, especially at pH 7.0 and 8.0, in which MBC was found only in the presence of 50% sucrose (625.0 mg/L and 1250.0 mg/L, respectively). Conversely, under acidic conditions, the increase of sucrose did not modify MIC values, and a negative impact on the bactericidal effect under higher sucrose concentrations was observed at pH 5. As RL showed greater bactericidal efficacy under conditions of acidic pH (pH 5.0), it is believed that the addition of high concentrations of sucrose alters the viscosity of the medium and consequently decreases the molecular mobility, which may be related to the antimicrobial activity observed under such pH value.

**Figure 1:**
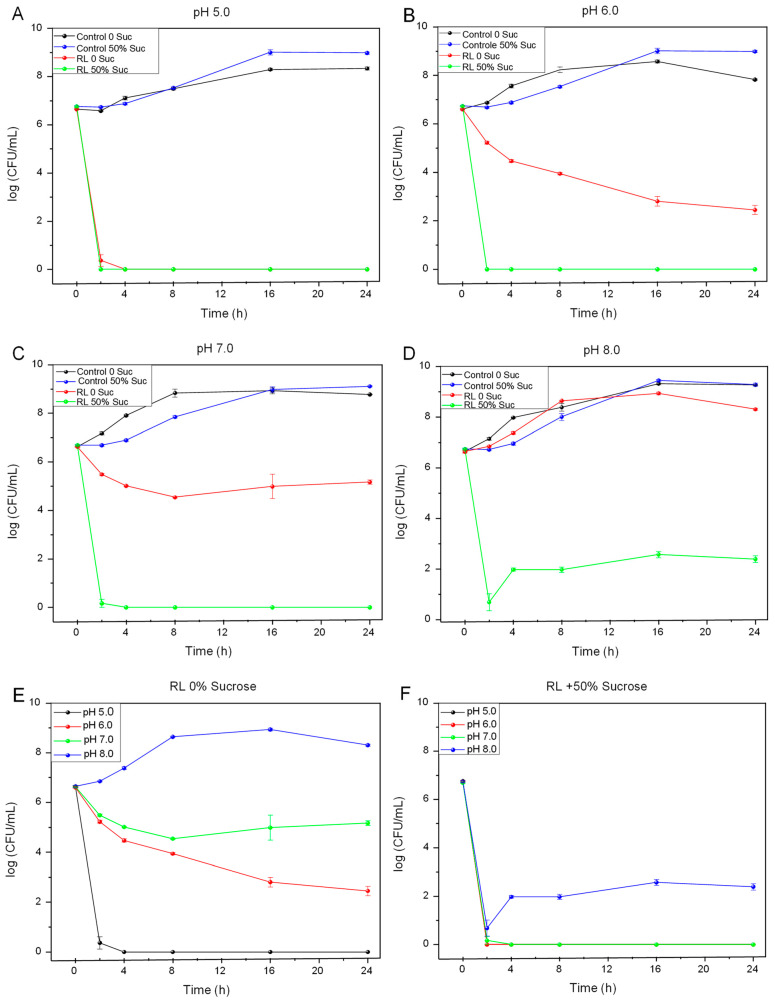
Growth of *L. monocytogenes* in the presence of 650 mg/L of RL with and without sucrose at (**A**) pH 5.0, (**B**) pH 6.0, (**C**) pH 7.0, (**D**) pH 8.0, and (**E**) RL at different pH values and (**F**) RL + 50% sucrose at different pH values.

In order to better evidence the effect of pH and sucrose on antimicrobial activity, it was decided to fix the concentration of RL (650 mg/L) and the highest concentration of sugar studied (50% *w*/*v*). Figure 1A shows that increasing the concentration of RL to 650 mg/L at pH 5 promoted comparable inhibition both in the presence and absence of sucrose, eradicating the population of viable cells in the first hours of incubation. At pH 6 and 7, the significant effect of the presence of sucrose on the antimicrobial activity of RL on *L. monocytogenes* was noted (Figure 1B,C). For pH 8, a reduction of approximately 6.9 log was observed compared with RL treatments in the absence of sugar (Figure 1D). In addition to the influence of pH, the presence of sucrose promoted an increase in the activity of RL on *L. monocytogenes*, especially at pH values 6 and 7 (Figure 1E,F), suggesting that the presence of this sugar has a positive effect on the action of RL, a fact that favors their application in food.

Rhamnolipids are anionic surfactants at neutral or alkaline pH, although, under acidic conditions, they behave as non-ionic surfactants due to the protonation of carboxylic groups present in their hydrophobic portion that have an acid dissociation constant (pKa) of 5.9 and 5.6 for mono-RL and di-RL, respectively [28,29]. Thus, when the pH is below this value, the polar groups of the RL remain protonated (COOH), being predominant in the non-ionic form, which, because it is free of effective charge, avoids electrostatic repulsion with the anionic groups of the cytoplasmic membrane, facilitating its action on the target cell structure [16]. At pH close to neutrality, or alkaline, the carboxyl group is mostly deprotonated and negatively charged (COO-), that is, in anionic form. The effect tends to increase according to the increase in pH value and may be related to the results observed for *L. monocytogenes*, where MIC values were higher as the pH of the medium increased (Table 1); in addition to the bactericidal effect observed at pH 5 and abundant growth at pH 8 (Figure 1E). These results corroborate the hypothesis that the presence of negative charges in the molecule hinders the interaction of the RL with the cell due to electrostatic repulsion. Moreover, our results are in agreement with previous reports showing that the antibacterial action of RL is pH-dependent [16,30]. Studies regarding surfactant-sugar interactions are scarce in the literature, and there is no report available about biosurfactant–sucrose interactions.

The presence of sucrose in the medium can result in different types of molecular interactions with the RL, for example, ion–dipole interactions, which occur between the hydroxyl groups (-OH) present in the sugar molecule and the carboxylic group of the BS in its ionized form (-COO-), in which the anion can attract the positive pole of the sucrose molecule. In contrast, RL molecules with their protonated carboxylic group (-COOH) can interact with the hydroxyl (-OH) present in sucrose molecules through hydrogen bonds. Dipole–dipole interactions between rhamnose units of RL and hydroxyl groups present in sucrose; induced dipole–dipole interactions, where the hydroxyl present in sucrose can induce the formation of a dipole moment in the nonpolar region of the RL, in addition to weak hydrophobic interactions, between the nonpolar domains of sucrose molecules and the surfactant, known as instantaneous dipole interactions can also occur [31].

The interactions between sucrose and RL can minimize the electrostatic repulsion between the negative charges present in the biosurfactant molecules (-COO-), especially at pH values above pka. In addition, bacterial cells may be more susceptible to the action of RL due to the stress caused by high concentrations of sugar in the medium [32]. Therefore, the sum of these factors could explain the greater sensitivity of bacteria to RL in the presence of sucrose.

### 3.2. Antimicrobial Activity in Biofilms

**Figure 2:**
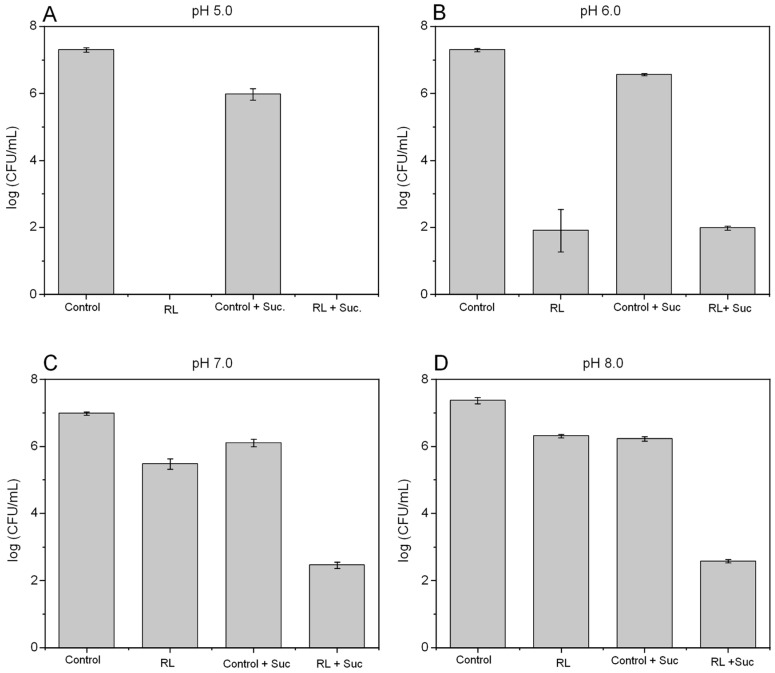
Effect of pH in the viability of *L. monocytogenes* biofilms treated with 650 mg/L of RL in the presence and absence of 50% sucrose. (**A**) pH 5, (**B**) pH 6, (**C**) pH 7, and (**D**) pH 8.

To check if the activity demonstrated by RL-pH-sucrose toward free *L. monocytogenes* cells can also be observed against sessile forms, similar tests were conducted within biofilms. Figure 2 shows that bacteria are able to establish biofilms under all pH tests and in the presence of 50% sucrose. It is possible to notice that at pH 5.0, both treatments, RL (650 mg/L) and RL–sucrose, resulted in the eradication of the *L. monocytogenes* population (Figure 2A). However, at pH 6.0, no significant differences were detected for the RL treatments with and without sugar (Figure 2B), both reaching around 5 log reduction relative to the controls. For pH 7.0 and pH 8.0, a greater reduction in the number of viable cells was observed with the RL–sucrose combination (3.6 log) compared with RL alone (1.5 and 1.0 log, respectively), suggesting better antimicrobial efficacy of the RL–sugar combination under these pH range (Figure 2C,D).

The MBIC and MBEC assay (Table 2) revealed a greater influence of the addition of solute on the antibiofilm activity of the RL at pH 7.0, in which the application of the RL alone showed resistance (>2500 mg/L), whereas the presence of sucrose resulted in bactericidal activity at 156.2 mg/L. At pH 5.0, although less expressive, the reduction in the MBEC value suggests that sucrose improves the bactericidal effect of the biosurfactant. It is worth noting that the RL–sucrose treatment showed bactericidal activity (MBEC 156.2 mg/L) at pH 7, conflicting with the data presented in Figure 2C, where the population was not eliminated under such conditions using 650 mg/L of RL. This difference may be attributed to the methods utilized to form the biofilms; in the peg lid technique, the biofilms were established around the pin that was submerged in the medium. Conversely, the polystyrene samples were deposited at the bottom of a plate filled with the medium; thus, settling can impact biofilm formation and architecture and, consequently, the antimicrobial action against it.

The RL–sucrose combination was more effective against planktonic cells compared with biofilms once the eradication of the population was observed in a wide range of pH values (Figure 1F). When growing in biofilms, cells are more resistant to stressful conditions, reducing the activity of antimicrobials, mainly due to the slow penetration through the polymeric matrix and to changes in the chemical microenvironment affecting cell growth [33]. The lower diffusion of the biosurfactant in the biofilm matrix may explain the greater efficacy of RL against the free-living cells.

The activity of biosurfactants in biofilms is associated with the weakening of interactions between cell-surface and bacteria-bacteria due to the reduction in surface and interfacial tension [34], thus favoring cell dispersion to the medium where they are more susceptible to the antimicrobials. In addition, disruption of the polymeric matrix can also facilitate RL molecules to penetrate and reach their cell targets [18]. The application of biosurfactants to biofilms is frequently described in the literature; however, to our knowledge, this is the first report showing the effect of pH and sugar in the activity of RL against bacterial biofilms.

Our results can be useful for developing RL-based strategies to control *L. monocytogenes* in food environments and products, for example, in the formulation of sweet food and fruit syrups where pH is generally acid, and also to mitigate biofilms in contact surfaces exposed to sugar-rich foods. In addition, activity demonstrated by RLs in the presence of high sucrose concentrations opens the perspective to their use in low a_w_ foods even alone and combined with other chemical or physical methods.

### 3.3. Impact of pH and Sucrose on Aggregation Behavior of RL

Some studies suggested that the antimicrobial activity of biosurfactants (BSs) would be related to the formation of micellar aggregates [35,36]. Critical micelle concentration (CMC) and dynamic light scattering (DLS) measurements are usually employed to predict the aggregation behavior of surfactants in solution. Moreover, it is known that the self-aggregation of amphiphilic molecules in solution depends on factors such as pH, ionic strength, and temperature, among others [37]; thus, the CMC and DLS of the RL solutions were evaluated in the range of pH and sucrose concentration utilized in the previous assays.

In the absence of sucrose, the CMC of the RL increases as the pH increases (Table 3). As previously discussed, the presence of negatively charged groups (pH > pKa) promotes greater repulsion between surfactant molecules, hindering the formation of micelles and consequently increasing CMC. The presence of sugar in the medium tends to attenuate the negative charge of the RL molecules when in ionic form (pH 6 to 8), which may be related to the reduction in CMC values for the RL–sucrose combination, especially between 5 and 10%. However, at higher concentrations, the addition of sugar can lead to increased viscosity of the medium, reducing the molecular mobility of BS [38]. In addition, the formation of diffuse sugar layers around the RL molecules could hinder their approximation for the formation of aggregates [39], leading to an increase in the observed CMC values.

Dynamic light scattering analysis of RL solutions under different working conditions revealed that at acidic pH (5 and 6), in the presence of 50% sucrose, the RL aggregates have size distribution similar to the control samples. At pH 7 and 8, the presence of sugar promoted an increase in the distribution amplitude of the molecular aggregates of RL, making the sample more heterogeneous in relation to the size of the particles In summary, there is a trend toward an increase in PDI values in the presence of sucrose, which is more evident for alkaline pH (Table 4). The RL solutions are characterized as polydisperse and heterogeneous, showing PDI values close to 0.5 at pH 7 [18,27], similar to the observed in this work.

**Figure 3:**
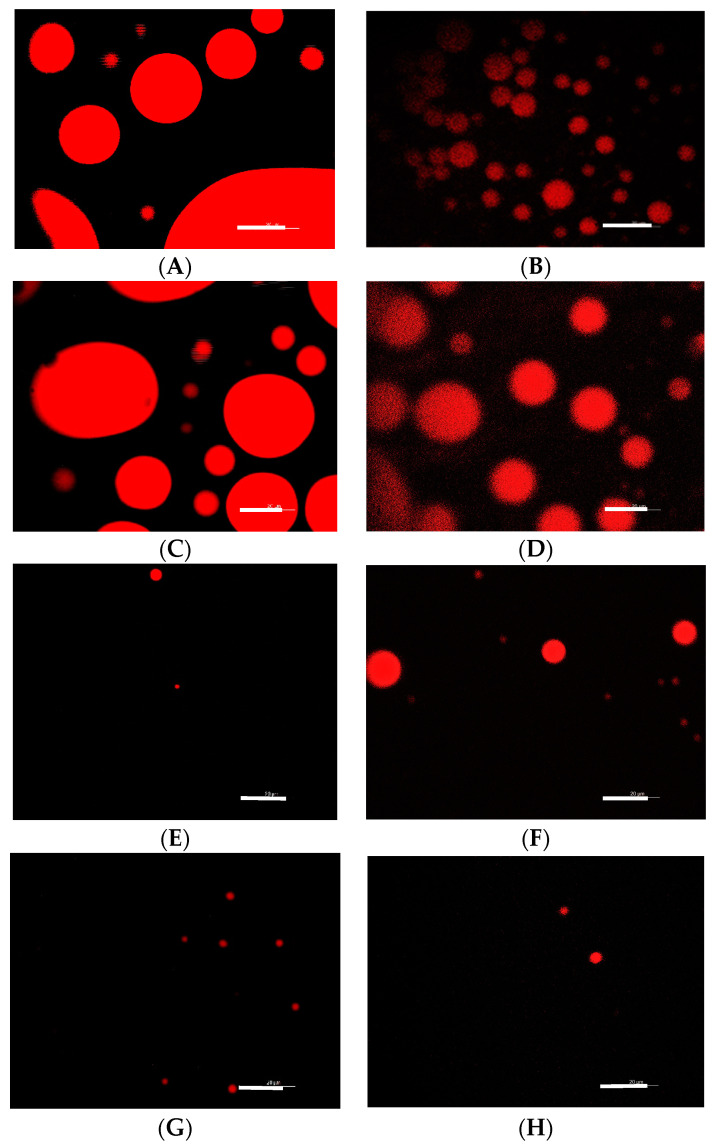
Fluorescence microscopy of the micellar structures of rhamnolipids formed in aqueous solution. (**A**) pH 5.0, (**B**) pH 5.0 + 5% sucrose, (**C**) pH 6.0, (**D**) pH 6.0 + 5% sucrose, (**E**) pH 7.0 (**F**) pH 7.0 + 5% sucrose, (**G**) pH 8.0, and (**H**) pH 8.0 + 5% sucrose. Scale bars correspond to 20 μm.

Fluorescence and Cryo-TEM Microscopies were performed to observe the self-assembly structures presented by RL under different working conditions. Fluorescence microscopy evidences the predominance of spherical micellar structures (Figure 3). The change in pH promoted changes in the size of the micellar structures of the RL, which are higher in acidic pH (Figure 3A,C) compared with neutral and alkaline (Figure 3E,G). The addition of sucrose at a concentration of 5% also promoted a change in the size of the aggregates with a similar tendency to the control (Figure 3B,D,F,H). Although it was not possible to observe the micellar structures formed in the presence of 50% sucrose due to the limitations of the technique, it was possible to notice that the presence of solute makes the micelles smaller when at acidic pH and apparently higher in the neutral/alkaline range when compared to RL without the presence of solute (control). These changes can be related to the data in Table 1, which shows a reduction in the MIC and MBC values for *L. monocytogenes* in the RL–sucrose combination at higher pH, suggesting that the antimicrobial activity of RL may be influenced by the type/size of the micellar structures present in solution.

**Figure 4:**
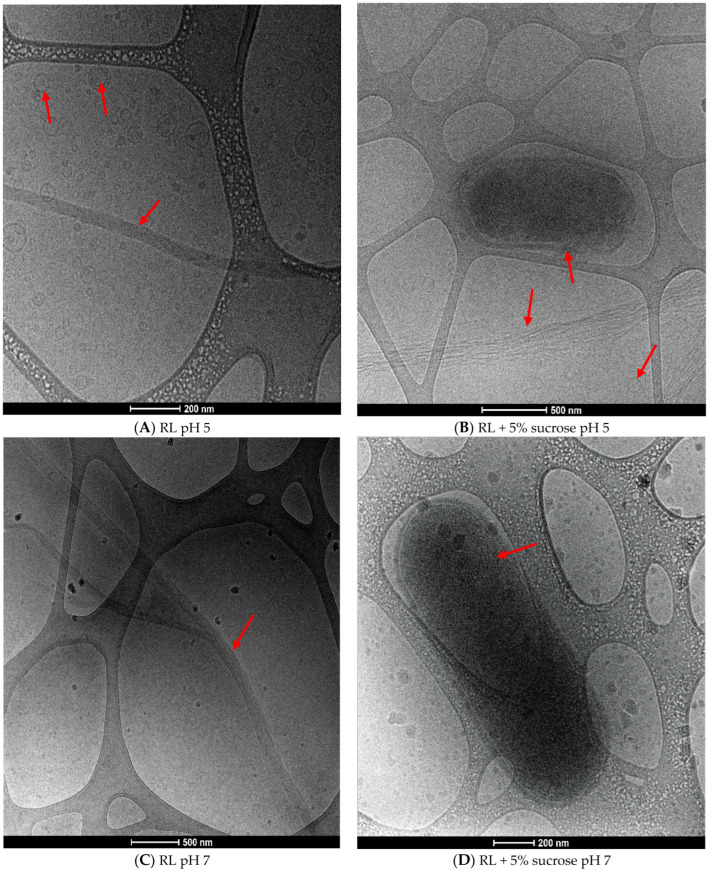
Cryo-TEM images of RL self-assembly structures at pH 5 in the absence (**A**), presence of 5% sucrose (**B**); pH 7 in the absence (**C**), presence of 5% sucrose (**D**). The red arrows highlight the predominant structures.

Cryo-TEM images show in detail the micellar structures formed by the RL molecules in solution (Figure 4). At pH 5, there is a predominance of spherical mono and by-layered vesicular structures. Moreover, elongated fiber-like molecular aggregates are present (Figure 4A). When sucrose is added, spherical structures together with lamellar-type aggregation are predominant (Figure 4B), and we hypothesize that the presence of the solute reduces interaction among RL fibers leading to a more unpacked structure resemble to lamellas.

The images corroborate this with fluorescence microscopy (Figure 3), which shows the prevalence of spherical structures at pH 5. There is also a tendency toward aggregation, with the formation of larger structures and multilayers, which could explain the reduction of CMC with 5% sucrose (Table 3) in addition to the increasing polydispersion of the system (Table 4).

At pH 7, the elongated fiber-like structures are predominant (Figure 4C). By contrast, when sucrose is present, spherical vesicles are observed together with multilayered structures (Figure 4D). It is noteworthy that RL did not present spherical structures at pH 7, corroborating Figure 3E, where limited fluorescence is evident, different from that observed in the presence of sucrose (Figure 3F). Probably, the elongated structures predominant at pH 7 are not adequately stained by the Nile red due to their shape/organization or by the limitation of the technique.

Elongated micellar structures (fibers, ribbons) have been observed by Cryo-TEM for biosurfactants containing two molecules of sugars in the polar portion, such as sophorolipids [40] and rhamnolipids [21]; however, their presence depends on the surfactant concentration, pH, ionic strength, temperature, and also the type and proportion of homologs in the RL mixture that can influence the micellar aggregation [41].

The amphiphilic character of biosurfactants is associated with their antimicrobial action since they can easily interact with phospholipids bilayers, causing membrane disruption and cell lysis [42,43]. In this sense, it is interesting to note that Cryo-TEM images showed bacterial cell that seems to be damaged by the surfactant with an apparent disruption of cell envelope (Figure 4B) and also a possible contact (fusion) with a multilayered structure (Figure 4D).

The antimicrobial action of RL is favored when there is a predominance of vesicular aggregates, as observed at pH 5. At pH 7, where fiber-like micelles are present, lower activity is also observed. However, the presence of sucrose induced the formation of lamellar/multilayered type structures, increasing antimicrobial activity against *L. monocytogenes*. The results suggest that the multilayered molecular aggregation that resembles the structure of the cytoplasmic membrane may favor the interaction and fusion of the RL with the bacterial membrane, enhancing their activity. However, further studies should be conducted to confirm this hypothesis. To our knowledge, this is the first report in the literature concerning the interaction of RL–sugar and its correlation with antimicrobial activity.

## 4. Conclusions

We demonstrated that the presence of sucrose can affect the antimicrobial activity of the RL biosurfactants against *L. monocytogenes*, even planktonic and biofilms. The addition of sugar improves RL action, especially under low acidic and neutral conditions. Moreover, the type/size of RL self-assembly structures was shown to be dependent not only on the pH but also on sucrose concentration. The presence of solutes and changes in pH levels influence not only the growth and persistence of bacteria but also their interaction with rhamnolipid micellar structures, suggesting a direct correlation between molecular aggregation and antimicrobial activity. These results open perspectives to the development of innovative methods for controlling *L. monocytogenes*, especially in sugar-rich or -low a_w_ foods and environments.

## Figures and Tables

**Table 1:** Influence of pH and sucrose concentration in the antimicrobial activity of RL against *L. monocytogenes*.

pH					% Sucrose					
	0%		5%		10%		25%		50%	
	MIC *	MBC **	MIC	MBC	MIC	MBC	MIC	MBC	MIC	MBC
5.0	19.5	78.1	19.5	78.1	19.5	78.1	19.5	312.5	19.5	625.0
6.0	39.0	>2500	39.0	>2500	39.0	>2500	39.0	1250.0	39.0	625.0
7.0	625.0	>2500	312.5	>2500	156.2	>2500	156.2	>2500	39.0	625.0
8.0	>2500	>2500	>2500	>2500	>2500	>2500	>2500	>2500	78.1	1250.0

* Minimal inhibitory concentration (mg/L); ** minimal bactericidal concentration (mg/L).

**Table 2:** Antimicrobial activity of RL and RL–sucrose against *L. monocytogenes* biofilms under different pH values.

pH	Treatment	MBIC * (mg/L)	MBEC ** (mg/L)
5.0	RL	19.5	78.1
	RL + 50% sucrose	19.5	19.5
7.0	RL	>2500	>2500
	RL + 50% sucrose	39.0	156.2

* Minimal biofilm inhibitory concentration; ** minimal biofilm eradication concentration.

**Table 3:** CMC (mg/L) values of rhamnolipid aqueous solutions under different pH and sucrose concentrations.

pH	% Sucrose				
	0	5	10	25	50
5.0	19.6	12.4	24.8	39.7	25.6
6.0	93.2	39.7	41.2	82.9	112.6
7.0	94.2	41.9	96.6	87.7	117.1
8.0	121.4	119.7	117.3	94.5	119.1

**Table 4:** Effect of pH and sucrose in polydispersity index (PDI) of rhamnolipids molecules in aqueous solutions.

% Sucrose	pH			
	5.0	6.0	7.0	8.0
0	0.421 ± 0.01	0.316 ± 0.01	0.428 ± 0.05	0.427 ± 0.01
50	0.533 ± 0.02	0.604 ± 0.01	0.573 ± 0.03	0.991 ± 0.01

## Data Availability

The original contributions presented in the study are included in the article, further inquiries can be directed to the corresponding author.
